# EUS‐Based Prediction of Variceal Recurrence After EVL Plus APC: Endohepatology Innovation Cannot Substitute for Portal Hypertension Standard of Care

**DOI:** 10.1111/den.70312

**Published:** 2026-10-19

**Authors:** Antoine Debourdeau, Lucy Meunier

**Affiliations:** ^1^ Department of Hepatogastroenterology, CHU Montpellier Université de Montpellier Montpellier France; ^2^ Department of Hepatogastroenterology, CHU Nîmes Université de Montpellier Nîmes France

**Keywords:** beta‐blockers, endoscopic variceal ligation, endosonography, esophageal and gastric varices, portal hypertension, secondary prevention

1

We read with interest the study by Tezuka et al. [1] demonstrating that perforating veins (PVs) detected by endoscopic ultrasonography (EUS) after endoscopic variceal ligation (EVL) combined with argon plasma coagulation (APC) independently predict esophageal variceal recurrence. While the competing risk framework and uniform protocol are methodological strengths, three limitations deserve consideration.

First, the study evaluates a treatment strategy not specifically recommended by current international guidelines while omitting the one that is. EVL combined with APC is not specifically endorsed by ESGE or Baveno VII guidelines; EVL alone remains the reference endoscopic treatment [2, 3]. Non‐selective beta‐blockers (NSBBs), prescribed to only 2/163 patients (1%), are guideline‐recommended first‐line therapy for secondary prophylaxis and routinely combined with EVL [2, 3]. This near‐total absence of NSBBs undermines external validity: in Western practice where NSBB use exceeds 60%–70%, hemodynamic conditions and recurrence kinetics differ substantially. Whether PVs retain predictive value in NSBB‐treated patients remains unknown.

Second, EUS was performed only after APC, without pretreatment assessment. Whether PVs represent a preexisting marker of advanced portal hypertension or persistent vessels reflecting incomplete mucosal obliteration—two mechanistically distinct entities—cannot be determined. The absence of paired pre/post‐treatment EUS data also precludes assessment of the protocol's effect on collateral vessel architecture.

Third, the multivariable model includes six covariates for only 37 recurrence events (events‐per‐variable ratio approximately 6), below the recommended threshold of 10. The reported sub‐distribution hazard ratio (SHR 3.97; 95% CI, 1.99–7.92) should be interpreted cautiously, and external validation is warranted before clinical translation.

Future studies should evaluate PV detection within a guideline‐adherent framework including systematic NSBB therapy and hepatic venous pressure gradient assessment to establish the incremental value of EUS‐based risk stratification.

## Author Contributions

**Antoine Debourdeau:** conceptualization, writing – original draft, writing – review and editing. **Lucy Meunier:** writing – review and editing, critical revision for important intellectual content.

## Funding

The authors have nothing to report.

## Conflicts of Interest

The authors declare no conflicts of interest.

## Linked Articles

This article is linked to Tezuka et al. paper. To view this article, visit. https://doi.org/10.1111/den.70132.

## Data Availability

Data sharing not applicable to this article as no datasets were generated or analysed during the current study.
